# Prevalence of tracheal collapse syndrome, congenital portosystemic shunts, or both in Yorkshire Terriers at one veterinary hospital

**DOI:** 10.1093/jvimsj/aalag094

**Published:** 2026-05-14

**Authors:** Chick Weisse, Sin Yin Kwok, Allyson Berent, Caroline Andy

**Affiliations:** Interventional Radiology and Endoscopy Service, Schwarzman Animal Medical Center, New York, NY, United States; Interventional Radiology and Endoscopy Service, Schwarzman Animal Medical Center, New York, NY, United States; Interventional Radiology and Endoscopy Service, Schwarzman Animal Medical Center, New York, NY, United States; Division of Biostatisitcs, Department of Population Health Sciences, Weill Cornell Medicine, New York, NY, United States

**Keywords:** congenital portosystemic shunts, prevalence, tracheal collapse syndrome, veterinary medicine, Yorkshire Terriers

## Abstract

**Objectives:**

The prevalence of tracheal collapse syndrome (CTCS) and congenital portosystemic shunts (CPSS) in Yorkshire Terriers is poorly characterized. It is conceivable that CPSS could affect the likelihood of CTCS development if the observed prevalence rate of both conditions is lower than the expected prevalence rate based on multiplication of the individual prevalence rates of each condition.

**Animals:**

Yorkshire Terriers diagnosed with CTCS, CPSS, or both at the Animal Medical Center (AMC).

**Methods:**

In this cross-sectional, single-institution study, medical records of Yorkshire Terriers were reviewed for patient date of birth, sex, weight, date of last visit, and reported diagnoses of CTCS, CPSS, or both. Medical records were reviewed to confirm the diagnosis of CTCS or CPSS. Observed and expected age-specific prevalence rates were compared within defined age groups.

**Results:**

A total of 11 061 Yorkshire Terriers were identified, with 7263 having data for age at last visit available. The observed prevalence of CPSS was 0.8% (93 dogs), severe CTCS was 6.7% (739 dogs), and both conditions were 0.05% (6 dogs). The median age at diagnosis was 2.7 years (interquartile range [IQR], 1.0-5.7) for CPSS and 10.0 years (IQR, 8.0-13.0) for CTCS. The observed age-specific prevalence rate of both conditions (joint) in each age group was similar to the expected joint prevalence rates in each age group.

**Conclusions:**

Further investigation is needed to confirm whether there is a link between CPSS and the development of CTCS in Yorkshire Terriers. Investigation into genetic and physiologic interactions is warranted.

## Introduction

Yorkshire Terriers are a popular breed predisposed to a range of congenital and acquired conditions, including tracheal collapse syndrome (CTCS) and congenital portosystemic shunts (CPSS).^1-3^ Tracheal collapse syndrome is a progressive disorder characterized by different forms of dynamic (traditional) or static (tracheal malformation) airway narrowing, frequently occurs in middle-aged to older dogs and can substantially impair quality of life.^3^ Congenital portosystemic shunts, a vascular anomaly that allows portal blood to bypass the liver, typically are manifested in young dogs with systemic signs of hepatic dysfunction. Although each condition has been studied independently, the potential coexistence and interaction between these conditions remain poorly understood. Yorkshire Terriers have been identified as the most common breed affected by both conditions.^1-3^

Recent studies have emphasized the presence of increased serum hyaluronic acid (HA) concentrations in dogs with CPSS because of decreased hepatic removal, and concentrations return to normal after shunt attenuation.^4,5^ Hyaluronic acid (HA), a glycosaminoglycan, is a key component of the extracellular matrix and plays a role in various physiologic processes, including tissue hydration, inflammation, and wound repair. It is also a critical component of hyaline cartilage, providing rigidity and stiffness along with other glycosaminoglycans (GAGs) and collagen in the tracheal hyaline cartilage rings.^6^ Dogs with dynamic CTCS experience degenerative changes in the tracheal hyaline cartilage, contributing to airway collapse.^7,8^ We have observed a low prevalence of dogs suffering from both CPSS and CTCS and hypothesize that there could be possible interactions between these 2 conditions. Although it seems unlikely that the development of CTCS in middle age would influence the presence of a CPSS, it is conceivable that the reverse could be true (ie, the presence of CPSS and resulting supraphysiologic serum HA concentrations could provide a protective effect against later degradation of the tracheal cartilage rings in CTCS). The interplay between these mechanisms raises intriguing questions about whether differences in age of onset and progression of these conditions provide a unique opportunity to study their interaction. We aimed to evaluate the prevalence of CTCS, CPSS, and both disorders in Yorkshire Terriers at a large referral hospital to investigate the presence of an association. Identifying potential interactions between these diseases may inform further investigation into these conditions.

## Materials and methods

### Study design and population

Our retrospective study reviewed all available medical records pertaining to Yorkshire Terriers from the Animal Medical Center (AMC), New York City. Patient medical records initially were reviewed for the mention of specific keywords including “shunt,” “tracheal collapse,” “collapsing trachea,” “tracheal malformation,” and “tracheal narrowing.” Subsequently, only dogs with confirmed diagnoses of CPSS, CTCS, or both based on a review of imaging reports and associated medical records were defined as prevalent cases in the study. Acceptable imaging for CPSS diagnosis included computed tomography (CT), ultrasonography, exploratory surgery, or radiographs documenting the presence of an ameroid constrictor or thin-film banding. Acceptable imaging for CTCS diagnosis included at least 1 lateral radiograph with tracheal lumen narrowing, or a tracheal stent visible on radiographs, CT, or tracheoscopy. Dogs diagnosed with different conditions or those with incomplete diagnostic data were classified as having neither condition. Tracheal collapse syndrome was considered confirmed if a 50% or more compromise of the tracheal lumen was identified on available imaging.

### Data collection

Data extracted included date of birth, sex, weight at time of diagnosis, date of last visit, and diagnoses of CTCS and CPSS. Diagnoses were confirmed based on clinical records, imaging studies, surgical reports, or a combination of these.

Age at diagnosis was calculated for both CPSS and CTCS. Observed age-specific prevalence rates of CPSS, CTCS, and both conditions were calculated based on the eligible population.

### Statistical analysis

Patients initially were classified into age groups of 1-4 years, 5-8 years, 9-12 years, and 13-15 years to facilitate examination of age-specific prevalence rates. These age groupings were chosen because of the very low number of patients with both CPSS and CTCS in the cohort. Assuming independence between CPSS and CTCS, the expected joint age-specific prevalence rate of having both CPSS and CTCS (for each defined age group) was calculated as the product of the individual age-specific prevalence rates (ie, observed CPSS age-specific prevalence rate multiplied by observed CTCS age-specific prevalence rate = expected joint [CPSS/CTCS] age-specific prevalence rate). The observed (actual) age-specific joint CPSS/CTCS number of prevalent cases (ie, cases with both conditions) and corresponding observed age-specific prevalence rates then were compared with the expected age-specific joint CPSS/CTCS number of prevalent cases and corresponding expected age-specific prevalence rates.

Dog characteristics including age and sex were reported by diagnosis category (confirmed or suspected CPSS and CTCS), disease severity category, and tracheal collapse type. Median and IQR were reported for continuous measures, and counts and percentages for categorical variables. Statistical differences between diagnosis groups were evaluated using the χ-squared test or Fisher’s exact test, as appropriate. A threshold of *P* < .05 was used to assess statistical significance.

## Results

A total of 11 061 Yorkshire Terriers were evaluated at the AMC during the study period, with age at last visit data available for 7263 dogs. Demographic characteristics of the study population are summarized in Tables 1-5. Median age for CPSS diagnosis was 2.7 years (IQR, 1.0-5.7) and median age for CTCS diagnosis was 10.0 years (IQR, 8.0-13.0; Tables 3 and 4). Dogs diagnosed with CPSS weighed significantly less than those with CTCS (median weight, 2.8 kg vs 3.2 kg; *P* < .001; Table 2). The overall mean and median weight of the entire cohort were 3.0 kg (±0.9 kg) and 3.3 kg (IQR, 2.4-4.6 kg), respectively (Table 1). For dogs with CTCS, a significant difference was observed in median age at diagnosis for the traditional type (11.0 years) compared with the malformation type (8.0 years; *P* < .001; Table 5). In addition, dogs with the malformation type weighed significantly less than traditional type patients (2.70 kg vs 3.30 kg, respectively; *P* < .001; Table 5). Diagnoses of CTCS were made significantly more often in male dogs (59%) compared with female dogs (41%; *P* = .004), but CTCS subtype was not significantly different based on sex (*P* = .10; Tables 4 and 5).

**Table 1:**
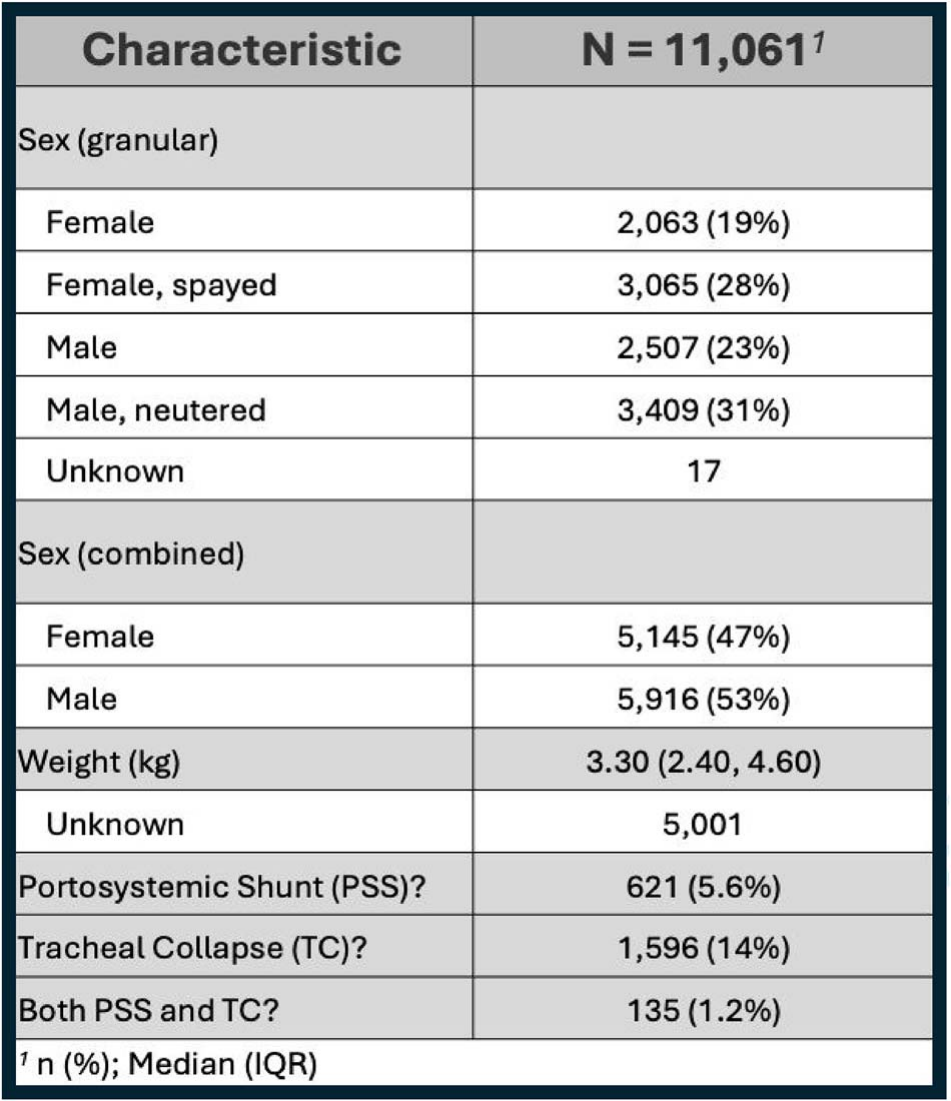
Demographic characteristics of study population. This table includes the following variables: sex, weight, number, and percent with PSS reported in medical records, number, and percent with TC reported in medical records, number, and percent with both reported in medical records.

**Table 2:**
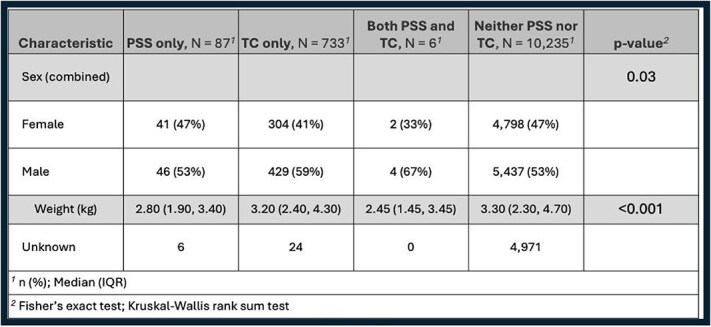
Prevalence of confirmed CPSS, CTCS, or BOTH diagnoses in Yorkshire Terriers including sex and weight comparisons. Not all confirmed cases had sex and weight information available.

**Table 3:**
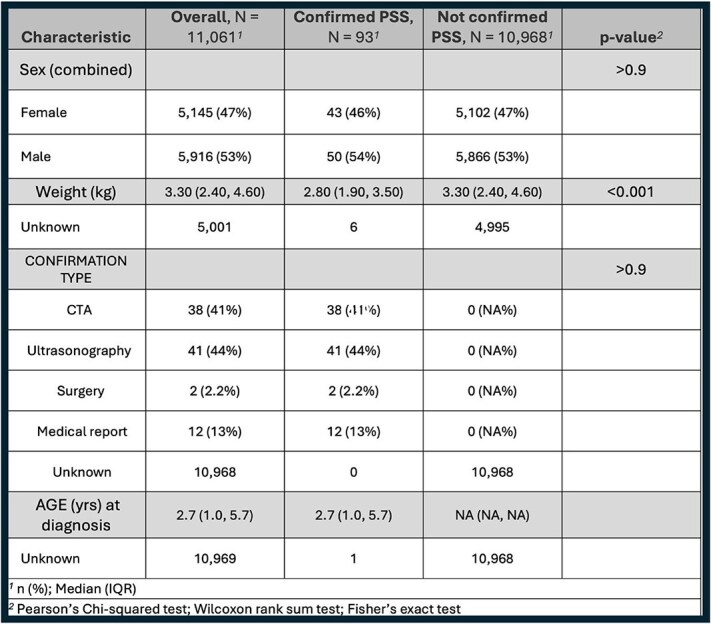
Prevalence of confirmed CPSS diagnoses in Yorkshire Terriers including sex, weight, method of confirmation, and age at diagnosis comparisons.

**Table 4:**
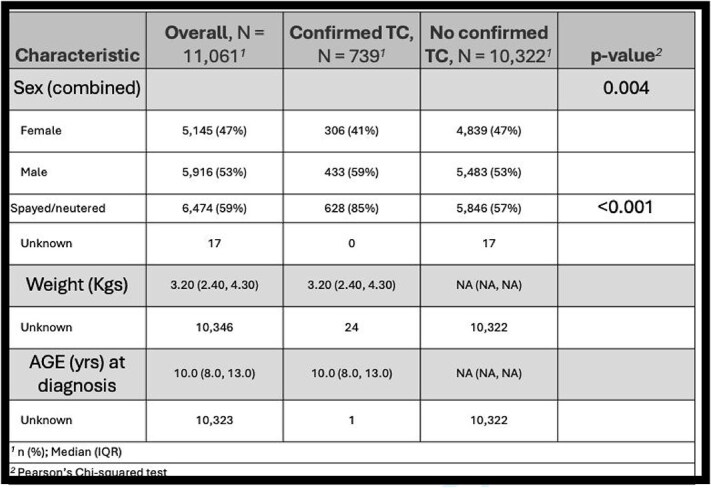
Prevalence of confirmed CTCS diagnoses in Yorkshire Terriers including sex, weight, and age at diagnosis comparisons.

**Table 5:**
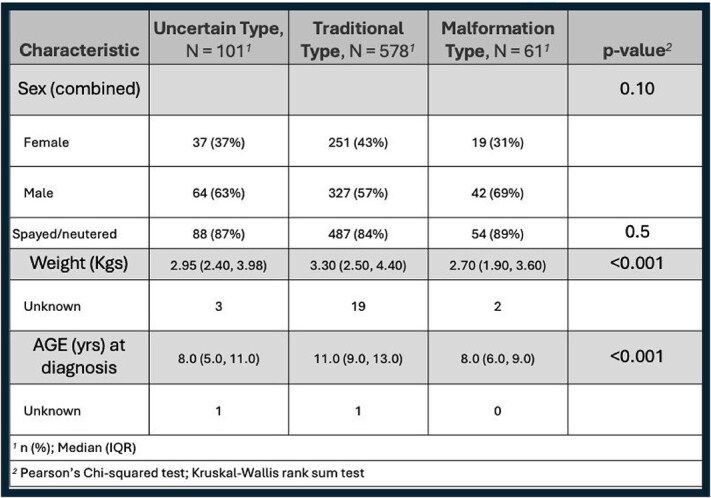
Prevalence of confirmed CTCS diagnoses in Yorkshire Terriers based on CTCS type (unclear, traditional type, or malformation type) including sex, weight, and age at diagnosis comparisons.

**Figure 1:**
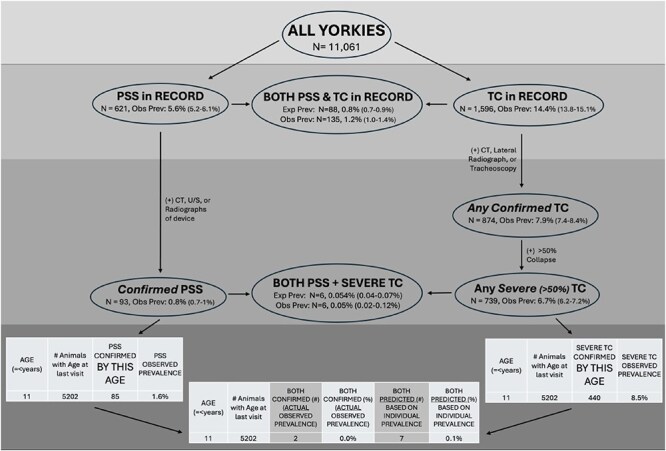
Incidence of PSS and tracheal collapse in Yorkshire Terriers. This diagram summarizes observed and expected prevalence of PSS, TC, and their overlap in 11 061 Yorkshire Terriers. The top section shows case counts and prevalence estimates for dogs with PSS, TC, or both in medical records. Diagnostic confirmation using imaging (CT, ultrasonography, radiography, or tracheoscopy) defines subsets with confirmed and severe cases (severe TC ≥ 50% collapse). The bottom panels display age-based incidence data: • left: confirmed PSS by age (8-11 years), • right: confirmed severe TC by age, and • center: co-occurrence of confirmed PSS and severe TC, with observed and predicted rates. This figure highlights age trends and the rare co-occurrence of severe forms of both conditions in Yorkies. Abbreviations: CT = computed tomography; PSS = portosystemic shunt; TC = tracheal collapse.

Ultimately, 93 dogs (0.8%) had confirmed CPSS, 739 dogs (6.7%) had confirmed CTCS, and 6 dogs (0.05%) had concurrent diagnoses of CPSS and CTCS. For the 6 dogs diagnosed with both conditions, CPSS was the first condition diagnosed, and all 6 dogs had traditional type CTCS (Figure 1; Table 6). When focusing on dogs aged 9-12 years, with 11 years being the median age of dynamic or traditional CTCS diagnosis, the expected prevalence was calculated to be 2 dogs with concurrent CPSS and CTCS based on observed rates of each individual condition (ie, expected joint rate = multiplication of the 2 observed individual rates). However, 3 dogs were observed in this age group (Table 7). The observed prevalence rate of concurrent conditions (0.15%) was higher than the expected prevalence rate of 0.10%. When looking specifically at 11 years of age, however, the observed prevalence rate of concurrent conditions (0%) was lower than the expected prevalence rate of 0.07% (Table S1).

**Table 6:**
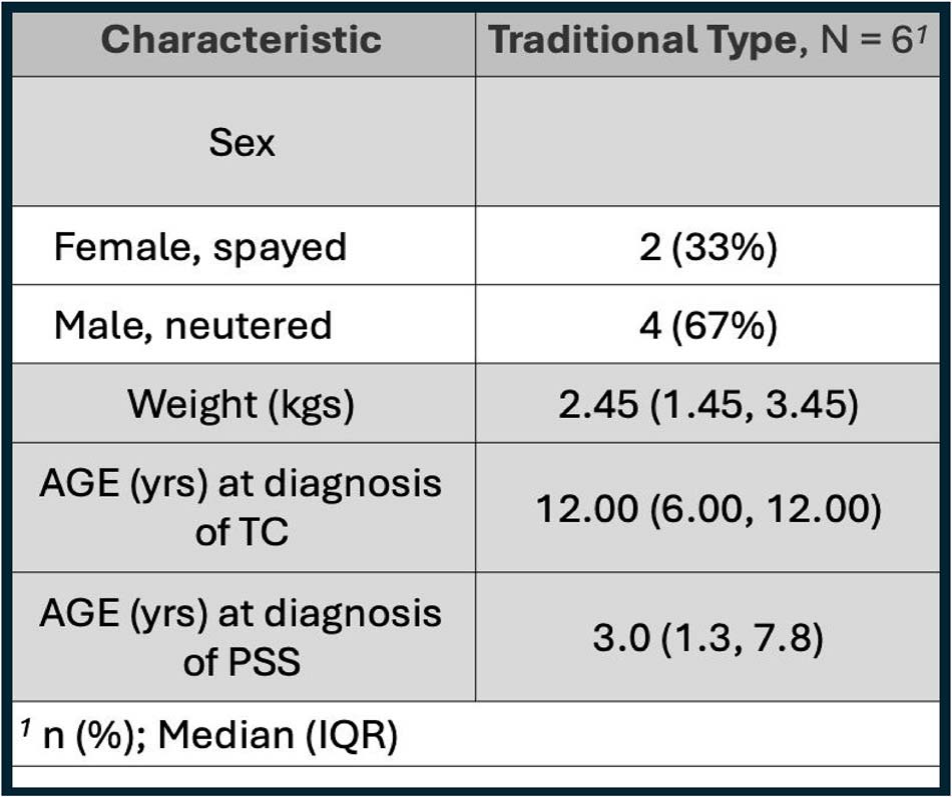
Prevalence of confirmed both (CPSS and severe [>50%] CTCS) diagnoses in Yorkshire Terriers including sex, weight, and age at diagnosis.

**Table 7:**
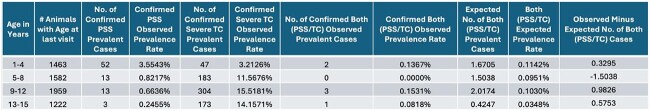
Age-specific observed and expected prevalences of CPSS, severe CTCS, and both conditions in Yorkshire Terriers in defined age groups.

## Discussion

Our study provides insight into the prevalence and potential interactions of CTCS and CPSS in a population of Yorkshire Terriers. This breed was chosen because of the relatively high prevalence of both conditions in the breed based on previous literature.^1,3,7^ Results identified here may not be applicable to other breeds, but it is interesting that Yorkshire Terriers are the only breed routinely listed in the top 5 breeds affected by either condition.^1,3,7^

Demographic characteristics of the current study population are similar to those previously reported. The median age of CPSS diagnosis of 2.7 years was similar to previous reports, including those for Yorkshire Terriers as well as other dog breeds, and no significant differences were identified between sexes.^1,2^ Age and sex distribution (male predisposition) for CTCS concur with previous studies, as does the likelihood of diagnosing the traditional (dynamic) form of CTCS in a slightly older population than the malformation subtype.^3,7^ The identified significant difference in dog weight between these subtypes may be associated with the age difference, chronicity of medical management, or other unidentified factors not assessed in our study.

Patient medical records initially were reviewed for mention of specific keywords including “shunt,” “tracheal collapse,” “collapsing trachea,” “tracheal malformation,” and “tracheal narrowing” to focus subsequent analysis of those records. This approach resulted in a far higher prevalence of these conditions than expected, and further review of those medical records ultimately led to the refined confirmed results reported above. Ultimately, 93 dogs (0.8%) had confirmed CPSS, 739 dogs (6.7%) had confirmed CTCS, and 6 dogs (0.05%) had concurrent diagnoses of CPSS and CTCS. A previous study in Yorkshire Terriers reported a CPSS prevalence of 3.6% based on a review of the Veterinary Medical Database.^2^ This observation demonstrates the difficulty in identifying the true prevalence of a disease in a population of dogs seen by different veterinary medical institutions. Any Yorkshire Terrier visiting the AMC may have satisfied the criteria for inclusion in the study. These individual patients did not all receive complete medical evaluations; it is therefore likely that some dogs were mistakenly identified as not having a CPSS or CTCS when in fact CPSS was present or CTCS would develop later. Once a dog was confirmed to have one or the other of these conditions, a more comprehensive evaluation subsequently may have been performed. Therefore, although the observed prevalence of each of these conditions individually may have been underestimated, it is likely that a patient suffering from both conditions would be more likely to be identified using a more comprehensive medical evaluation. This situation would result in underestimating the observed prevalence of each condition and therefore underestimating the expected prevalence of both conditions jointly. Alternatively, the temporal separation in disease onset may decrease the likelihood of concurrent diagnoses, because dogs with CPSS could experience substantial health challenges in early life that might preclude the later development ofCTCS.

One of the complicating factors of conducting our study was that these conditions often can be diagnosed later in life, particularly CTCS. Therefore, until a dog reaches a certain age, if it has not yet been diagnosed with CTCS, it is still possible that it will develop the disease later in life. For this reason, we chose not to look primarily at observed prevalence and expected combined prevalence of these conditions in the total cohort of all ages because doing so would likely introduce age bias. Instead, we defined age groups and examined the age-specific observed and expected prevalence rates of each of these conditions, and the joint prevalence rate, in these age groups (Table 7). Although evaluating age-specific prevalence at each year of life is difficult because of the low number of observed cases with both CPSS and CTCS, these data are provided in Table S1.

If some connection between these 2 conditions exists, it is unlikely that the development of CTCS later in life influences the presence of CPSS. However, CPSS could have an effect on hyaline cartilage early in the patient’s development. For the 6 dogs ultimately diagnosed with both conditions, CPSS was the first condition diagnosed. Unfortunately, the fact that only 6 patients were identified precludes any meaningful statistical analysis of these results. Interestingly, all 6 patients confirmed with both conditions suffered from the traditional (dynamic) CTCS subtype. Although it is the most common type of CTCS, occurring in 71.3%-91.8% of dogs with CTCS,^9^ if the underlying disease was related to dysregulated hyaline cartilage production, one would expect none of the patients to have the malformation type CTCS, as was observed here. Tracheal malformations, including those involving fixed, static structural deviations or cartilage anomalies, are less common compared with the traditional form of tracheal collapse. Traditional tracheal collapse is predominantly characterized by cartilage degeneration. Notably, all dogs with concurrent CTCS and CPSS in our study had traditional tracheal collapse rather than structural malformations, further supporting a potential role for HA in influencing the integrity of the tracheal cartilage.

Because the traditional subtype of CTCS is diagnosed later in life (median age, 11 years in our study), analysis was performed to compare expected and observed prevalence of both CPSS and CTCS in defined age groups to correct for differences in condition prevalence over the lifespan of the dogs. The expected prevalence for dogs aged 9-12 years was calculated to be 2 dogs with concurrent CPSS and CTCS based on observed rates of each individual condition in this age group but 3 dogs with both conditions were observed in this age group. One interpretation of this observation is that there is no evidence in the examined population that the observed prevalence of joint disease is meaningfully different from the expected prevalence of joint disease under the assumption of independent events.

Although the observed and expected results were not meaningfully different between each defined age group, the lower than expected prevalence of concurrent diagnoses at 11 years (Table S1) raises questions about potential protective mechanisms or interactions between the diseases. The possibility that supraphysiologic HA concentrations during development provide long-term protective effects against tracheal cartilage degeneration remains an area for further exploration.

The pathogenesis of traditional (dynamic) tracheal collapse involves hypocellular hyaline cartilage with decreased amounts of glycoproteins and GAGs.^7,8^ Decreases in GAG, collagen, and water content lead to the flaccid, weakened, and flattened nature of the cartilage rings, stretching and weakening the dorsally attached trachealis muscle.^10^ Developmentally, tracheal rings initially possess compliance and flexibility in neonates that gradually stiffen and become less compliant with age in order to tolerate greater airway pressures in the growing animal.^6^ Compositional changes in the tracheal cartilage that occur with increasing age include a gradual increase in the width and amount of cartilage present as well as thickening of the trachealis muscle.^6^ Hyaluronic acid has been associated with increased proteoglycan synthesis, anti-inflammatory properties, and cartilage repair and tissue remodeling.^10^ It is conceivable that supraphysiologic HA concentrations could contribute to early effects on tracheal hyaline cartilage composition during early development.

Recently, serum HA concentrations have been used both to diagnose the presence of CPSS in dogs and to assess response to treatment.^4,5^ Hyaluronic acid is produced and found throughout the body and is primarily removed from the blood by endothelial cells of the hepatic sinusoids.^4^ Should dogs with CPSS have a decreased observed prevalence of CTCS, based on the observed prevalence of each of these individual conditions, HA concentrations could be implicated. Of course, there could be many other reasons for an interaction between these 2 conditions that were not explored in our study.

Our study had several limitations inherent to its cross-sectional design. Reliance on medical records introduces potential biases related to data completeness and diagnostic accuracy. In addition, the single-institution setting may limit the generalizability of findings to other populations or geographic regions. Full diagnostic evaluations were not performed on all dogs because many were presented to the hospital for unrelated reasons. This factor likely led to an underestimation of the true prevalence of these conditions, and we may have missed additional cases of concurrent CPSS and CTCS.

Future research should investigate whether targeted interventions for CPSS could influence the subsequent development of CTCS. Longitudinal studies tracking the health trajectories of Yorkshire Terriers diagnosed with either condition could provide valuable insight into their natural history and potential interactions. Investigation of CTCS prevalence in untreated CPSS dogs, or in those with portal vein hypoplasia without portal hypertension that are typically managed conservatively, would be interesting because a decreased prevalence of CTCS in this population subset would support the possible protective effect of CPSS in these dogs. Finally, genetic studies could shed light on shared or opposing pathways that influence disease risk in this breed. The role of HA as a potential protective or risk-modifying factor should also be explored in greater detail to elucidate its contribution to disease progression. Veterinarians managing Yorkshire Terriers should remain vigilant for both conditions, particularly in age appropriate populations.

## Supplementary Material

aalag094_New_Supplementary_Table_1

## Off-label antimicrobial declaration

The authors declare no off-label use of antimicrobials.

## Institutional animal care and use committee or other approval declaration

The authors declare no institutional animal care and use committee or other approval was needed.

## Abbreviations

AMC: Animal Medical Center
CPSS: congenital portosystemic shunts
CTCS: canine tracheal collapse syndrome
GAG: glycosaminoglycan
HA: hyaluronic acid
