# Supplementary figures and images for: Prevalence of tracheal collapse syndrome, congenital portosystemic shunts, or both in Yorkshire Terriers at one veterinary hospital
Source: J Vet Intern Med. 2026 May 14;40(3):aalag094. doi: 10.1093/jvimsj/aalag094 (PMC13173432; doi:10.1093/jvimsj/aalag094)

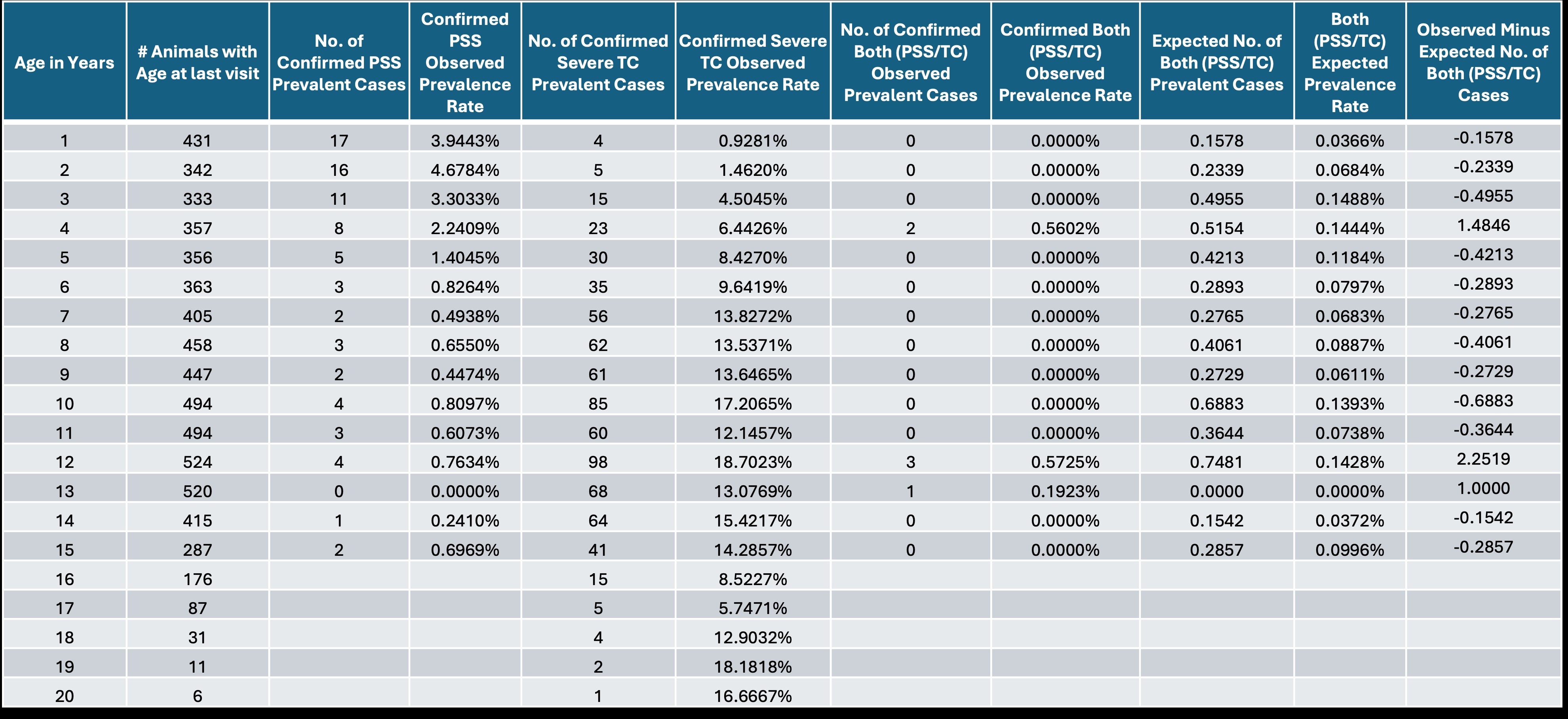

Supplement: aalag094_New_Supplementary_Table_1 [file aalag094_new_supplementary_table_1.jpeg]
